# Multivariate temporal modeling of crime with dynamic linear models

**DOI:** 10.1371/journal.pone.0218375

**Published:** 2019-07-03

**Authors:** Nathaniel Garton, Jarad Niemi

**Affiliations:** Department of Statistics, Iowa State University, Ames, IA, United States of America; National Taiwan University, TAIWAN

## Abstract

Interest in modeling contemporary crime trends, a task that has historically been considered valuable to the public, researchers, and policymakers, is resurging. Advancements in criminology have made it clear that understanding crime trends necessarily involves understanding trends in how likely individuals are to report crimes to the police, as well as how likely the police are to accurately record those crimes. In this paper, we use dynamic linear models to simultaneously model the time series for several crime types in order to gain insight into trends in crime and crime reporting. We analyze crime data from Chicago spanning 2007 through 2016 and show how correlations in the way crime trends evolve may contain information about drivers of crime and crime reporting. We provide evidence of substantial differences in the relationships between the trends of crimes of different types depending on whether crimes are violent or nonviolent and whether or not crimes are tracked in the FBI’s Uniform Crime Report.

## Introduction

In the 2018 *Annual Review of Criminology* Baumer et al. state in their paper, “Bringing Crime Trends Back into Criminology: A Critical Assessment of the Literature and a Blueprint for Future Inquiry,” a surprising fact: that “the study of crime trends is not part of mainstream criminological theory or research” [1]. However, as one might expect from the title of their paper, they argue that it ought to be. And, though there has been some research on contemporary crime trends, with respect to this research the authors note a number of issues including little attention to differences in crime trends based on crime type. They also note that crime trends can depend heavily on the data source, and they cite an example of how police data from the Uniform Crime Reports (UCR) has, in the past, differed from that of data from the National Crime Victimization Survey (NCVS): a difference that has previously been noted and analyzed [2]. Additionally, they describe a continued interest in comparing crime trends in multiple cities or regions of cities: an interest that has also been previously addressed [3, 4].

Additionally, while there has been some research into trends in the reporting of crime at the national level [5], to our knowledge there has been relatively little study on crime reporting specifically in the city of Chicago. Yet, recent questions have been raised with regard to the integrity of reported crime in Chicago. *The Economist* published an article in 2014 calling for skepticism when interpreting the sharp declines in reported crime in Chicago seen around 2013 [6]. Reasons for this skepticism include evidence published in *Chicago* magazine that police officers were under pressure to under-report or downgrade certain types of crime [7]. Of particular interest were those crimes which are part of the FBI’s UCR. In both articles, one possible suggestion for how misreporting might have taken place was that burglaries, tracked in the UCR, could have been misclassified as criminal trespasses, a crime not similarly tracked. Criminal trespasses are one of the types of crime reported in our dataset, and the question of whether trends in criminal trespasses are negatively correlated with burglaries can be answered probabilistically in the DLM framework we propose. More details about the UCR can be found on the FBI’s UCR website [8].

Of multivariate analyses of multiple crime types that have been done, there has been some focus on understanding spatial associations between different types of crime or comparing spatio-temporal patterns across crime types using visualization tools [9, 10]. Other temporal multivariate crime analyses, like ours, have examined the dynamic relationship of serious crimes with mild crimes [11–13], but the goals in those analyses were largely to test the “broken windows theory” that mild and visible crime encourages more serious crimes. In this paper, we propose the novel use of state-space models, specifically dynamic linear models (DLMs), not only as a tool to simultaneously model multiple crime time series, but as a natural way to quantify similarities and differences between crime trends. DLMs are generalizations of ARIMA models [14]. While DLMs have often been used in a forecasting context, we take advantage of them for the purpose of inference on historical crime trends and, critically, trend correlations. Using recent data from Chicago, we show how clear relationships between crimes emerge based on the categorization of crimes as either violent and tracked in the UCR, nonviolent and tracked in the UCR, or nonviolent. Thus, we take aim at one of the the several shortcomings of modern analysis of crime trends, which, according to [1], has gained little attention. Two significant differences between our analysis and the other multivariate temporal crime analyses are that we examine contemporaneous, rather than lagged, trend correlations, and rather than focus on testing the “broken windows theory” by looking for *positive* correlations between mild and serious crimes, we examine the hypothesis that misreporting of serious crimes as mild crimes has yielded an artificial change in the trends of serious crimes by looking for *negative* correlations between burglaries and criminal trespasses. While it remains possible that crime in Chicago may, indeed, have been underreported in recent years, but it seems unlikely that burglaries have been misclassified as criminal trespasses to any significant extent. We stress, however, that many of the other issues raised by Baumer et al. could be addressed through this methodology, as many extensions and variations on the relatively simple models presented here are possible.

## Data

The dataset used in our crime analysis is publicly available and consists of all reported crime in Chicago going back to 2001, which can be found on the city of Chicago’s website [15]. One of the intended uses for these data is for academic research, and we have complied with the terms of use of the data as specified on the city of Chicago’s website. We take a moment to re-emphasize that reported crime, clearly, is not the same as crime. Any conclusions about multi-year trends observable in the data do not necessarily correspond to multi-year trends in crime, but they may have to do with trends in crime reporting. Therefore, while for convenience we often refer to reported crimes simply as crimes, caution should be taken when interpreting our results. In either case, however, we find results about trends in reported crime and the relationships between these trends as being interesting and, hopefully, potentially useful.

This dataset provides a rich set of information, allowing for much more sophisticated analyses than are presented here. Along with other information, the date, type, and approximate (anonymized) location of each individual crime is recorded. In order to address the issues raised by *The Economist* and *Chicago* magazine, we need to at least include data for burglaries and criminal trespasses since 2011: just before the years when the steep declines in crimes were noticed. Further, the timescale at which these declines were noticed was that of years. Therefore, we aggregated the data by months, as this allowed for a detailed analysis of the trends over this time period while avoiding so many time periods that computation became troublesome. In order to provide additional context for the crime declines already mentioned, we included four additional crime types: robberies, assaults, narcotics crimes, and motor vehicle thefts. All off these additional crime types were included in the FBI’s UCR except for narcotics [8]. Of the crimes reported in the UCR, robberies and assaults are considered violent crimes, while burglaries and motor vehicle thefts are considered property crimes [8]. Thus, the crimes that we analyzed could be grouped into one of three categories: violent and recorded in the UCR, nonviolent and recorded in the UCR, or nonviolent and not recorded in the UCR. Additionally, we included data from January 2007 through December 2016. Including this additional data provides some points of reference for the patterns and relationship that we observe with respect to burglaries and criminal trespasses.

**Fig 1:**
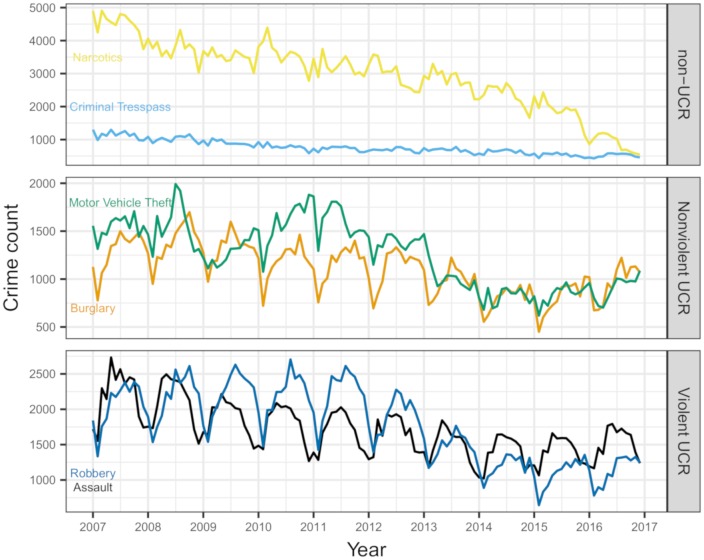
Monthly aggregated counts of six types of reported crimes in Chicago from January 2007 through December 2016.

Fig 1 plots monthly crime counts from all six crime types from 2007 through 2016. Monthly crime counts range from about 500 to 5000, with assaults, burglaries, robberies, and motor vehicle thefts often reaching similar numbers but with narcotics substantially exceeding the others, especially in years 2007-2014. Similarly, seasonal trends are obvious and seemingly account for substantial variability within assaults, robberies, and burglaries, but seasonality is less obvious for the narcotics, motor vehicle theft, and criminal trespass time series, where there appears to be much more random variability. Larger, multi-year trends do seem to exist for all crime types, with reported narcotics crime apparently sharply declining over the 10 year span. The remaining crime types do seem to more or less decline over time, but the nature of this decline varies. For example, robberies don’t appear to decline at all from roughly 2007-2011, at which point a sharp decline begins, which then levels out around 2014. Alternatively, assaults seem to decline much more slowly and consistently from 2007-2014, at which point they either level out or even begin to increase again.

Table 1 provides correlations between each pair of crime types. Most of the correlations exceed 0.5, and several are approximately 0.9. For example, the correlation between robberies and burglaries up to two decimal places is 0.90, and the correlation between motor vehicle thefts and burglaries is approximately 0.88. This provides some immediate evidence that temporal patterns in crime were similar between many of the crimes examined here and helps to motivate the desire to perform a multivariate analysis.

**Table 1:** Correlations of monthly crime counts from January 2007 to December 2016 in Chicago between burglaries, robberies, assaults, narcotics, motor vehicle thefts, and criminal trespasses.

	Burglary	Robbery	Assault	Narcotics	MVT	Trespass
Burglary	-	0.90	0.68	0.64	0.88	0.71
Robbery	0.90	-	0.68	0.38	0.76	0.63
Assault	0.68	0.68	-	0.51	0.61	0.79
Narcotics	0.64	0.38	0.51	-	0.62	0.80
MVT	0.88	0.76	0.61	0.62	-	0.68
Trespass	0.71	0.63	0.79	0.80	0.68	-

## Seemingly unrelated time series equations

The general class of models we will consider are called seemingly unrelated time series equations (SUTSE). Any ARIMA model can be expressed as a DLM [14, section 3.2.5]. In particular, an ARIMA representation as a DLM will have an error variance of zero, i.e. $\sigma_{i}^{2}=0\;\forall \;i$. SUTSE models are defined through two equations called the observation equation and the evolution, or state, equation. First, define *Y*_*c*,*t*_ for *c* ∈ {1, …, *C*} and *t* = 1, …, *T* to be the log of the number of reported incidents of crime type *c* in month *t*. Then we assume *Y*_*t*_ = (*Y*_1,*t*_, …, *Y*_*C*,*t*_)^⊤^ = *Fθ*_*t*_ + *ϵ*_*t*_, where *F* is a *C* × *p* matrix, *θ*_*t*_ is a *p* × 1 vector of latent states, and *ϵ*_*t*_ is a *C* × 1 vector of Gaussian errors with mean 0 and covariance matrix Σ_*ϵ*_. We have six crime types (*C* = 6), and therefore Σ_*ϵ*_ is a 6 × 6 matrix. The state equation we consider is given by *θ*_*t*_ = *Gθ*_*t*−1_ + *δ*_*t*_, where *G* is a matrix of dimension *p* × *p*, and *δ*_*t*_ is a *p* × 1 vector of (possibly degenerate) zero mean Gaussian random variables with covariance matrix Σ_*δ*_. It is worth noting that some elements of the *δ*_*t*_ vector may always be zero. This indicates that those elements of *θ*_*t*_ do not evolve over time. The evolutions, *δ*_*t*_, and the observation errors are assumed independent of each other. Thorough treatments of SUTSE models can be found in [16] and [14]. For clarity, we first introduce a simple model which will provide a platform for us to develop our final model that will allow us analyze crime trends and their correlations.

A chief goal in modeling these time series is to extract macroscopic trends absent of seasonal effects. A common way to model trends in a DLM framework is through a local linear trend model [14, 16]. We define the local linear trend model with independent evolutions and errors next. First, define *θ*_*c*,*t*_ to be the 2 × 1 state vector at time *t* for crime type *c*, and let *θ*_*t*_ denote the concatenation of state vectors for all crime types at time *t*. This implies that *θ*_*t*_ is a 12 × 1 vector. In order to define the distribution of *δ*_*t*_, we first define ${\tilde{\delta }}_{t}$ to be a 6 × 1 vector such that ${\tilde{\delta }}_{t}\overset{iid}{∼}N_{6}\left(0,\text{diag}\left(\gamma_{1}^{2},\gamma_{2}^{2},...,\gamma_{6}^{2}\right)\right)$. Then, define $\delta_{t}=[I_{6\times 6}⊗{\left(0,1\right)}^{⊤}]{\tilde{\delta }}_{t}$. This implies that *δ*_*t*_ is a 12 × 1 vector. Also, let $\epsilon_{t}\;\overset{iid}{∼}\;N_{6}\left(0,\text{diag}\left(\sigma_{1}^{2},\sigma_{2}^{2},...,\sigma_{6}^{2}\right)\right)$. Then, the remainder of this model is specified through the *F* and *G* matrices:

$$F=I_{6\times 6}⊗\left(1,0\right)\;\text{and}\;G=I_{6\times 6}⊗[\begin{array}{cc} 1 & 1\\ 0 & 1 \end{array}].$$ (1)

Then, the model can be written as above, namely, *Y*_*t*_ = *Fθ*_*t*_ + *ϵ*_*t*_ and *θ*_*t*_ = *Gθ*_*t*−1_ + *δ*_*t*_.

While the above model can be used for trend modeling, it cannot be used to do inference on the correlations between trends. This is because the evolutions, ${\tilde{\delta }}_{t}$, and the errors, *ϵ*_*t*_, are assumed to be independent. This independence assumption essentially means that doing this analysis is the same as doing six univariate analyses: one for each crime type. In order to do our desired inference on the evolution (and hence trend) correlations, we must consider, instead, a truly multivariate model. We can accomplish this by allowing for dependence of both the residual errors as well as the state evolutions. This is done in our case by allowing the covariance matrices for *ϵ*_*t*_ and ${\tilde{\delta }}_{t}$ to be unstructured. Stated mathematically, $\epsilon_{t}\overset{iid}{∼}N_{6}\left(0,\Sigma_{\epsilon }\right)$, ${\tilde{\delta }}_{t}\overset{iid}{∼}N_{6}\left(0,\Sigma_{\tilde{\delta }}\right)$. In this way, we allow for any possible collection of correlations between pairs of crime types. The dependencies between states or between observations carry different interpretations, but both may be of interest. Dependence of the residual errors relates deviations from the mean of the data model of different crime types to each other, whereas dependent evolutions relate deviations in the modeled trend of each crime type from a strictly linear trajectory to each other. Dependence of the residual errors will depend on spurious month-to-month influences, whereas dependence of the state evolutions will capture deeper relationships in long-term crime trends.

This interpretation of dependent evolutions should help to clarify how inference on the evolution correlations can help to shed light on the specific suggestion in the article from *The Economist* that declines in reported burglaries may be the result of misreporting as criminal trespasses. If such misreporting were taking place, then we would expect the trend in burglaries to decrease as the trend in criminal trespasses increased. This simultaneous change in trends for burglaries and criminal trespasses would result in negatively correlated evolutions. However, one would not necessarily need to see either an absolute increase in reported criminal trespasses or an absolute decrease in reported burglaries, as was reported in the case of New York, in order for evidence of this reporting issue to be seen. For example, if criminal trespasses consistently declined from 2007 to 2012, but the rate of decline then flatlined or slowed, this could be evidence of misreporting if, at the same time after 2012, reported burglaries declined more than was typical in 2007 to 2012.

The above model allows us to smooth the time series and to make inference about the dependencies in the trends as well as residual errors, but including an annual seasonal component should allow for a more precise estimate of the trends, and hopefully of correlation parameters. To represent seasonality, we chose to utilize the Fourier-form seasonal model [14]. Expressing seasonality this way is an alternative to parameterizing monthly seasonal effects with dummy indicator variables. An advantage to this parameterization is that it potentially allows for a sparser representation of seasonality. The Fourier-form seasonal model is defined in terms of periodic functions called harmonics and complementary functions called conjugate harmonics. The number of harmonics, *q*, determines the flexibility of this representation to describe seasonal patterns. The maximum possible *q* is determined by the period, *s*, which is the number of times after which the seasonal pattern repeats. In our case *s* = 12, and the maximum possible number of harmonics is *s*/2 = 12/2 = 6. Note that if we use *q* = 6 harmonics, we will arrive at the same representation of seasonality if we were to use 11 dummy month variables plus an intercept term. A given harmonic-conjugate harmonic pair arises for a given Fourier frequency *w*_*j*_ = 2*πj*/*s*, and *j* = 1, 2, …, *s*/2. Each of these harmonic-conjugate pairs occupies two of the 12 − 1 = 11 degrees of freedom possible to fully characterize the annual seasonality for a given crime type. Thus, at most six harmonics and five conjugate harmonics can be included in the model per type of crime. For crime type *c* and *j* = 1, …, 6, define the *j*-th harmonic to be *ξ*_*c*,*j*_(*t*) = *ζ*_*c*,*j*_ cos(*tw*_*j*_) + *η*_*c*,*j*_ sin(*tw*_*j*_). Then, for *j* = 1, …, 5 define the *j*-th conjugate harmonic to be $\xi_{c,j}^{*}\left(t\right)=-\zeta_{c,j}\;\text{sin}\left(tw_{j}\right)+\eta_{c,j}\;\text{cos}\left(tw_{j}\right)$. Having defined the harmonic and conjugate harmonic functions, the final model, which includes seasonality, we will call the dependent linear trend model with seasonality. Defining this model requires modifications to the *θ*_*t*_ vectors, the *F* and *G* matrices, as well as the matrix that we must multiply ${\tilde{\delta }}_{t}$ by in order to get *δ*_*t*_. First, if *q* < 6, then *θ*_*t*_ is a (12 + 12*q*) × 1 vector. Also, *δ*_*t*_ becomes $\delta_{t}=[I_{6\times 6}⊗{\left(0,1,0,0,...,0\right)}_{(2+2q)\times 1}^{⊤}]{\tilde{\delta }}_{t}$. It will also be useful to define some additional matrices in order to specify *G*. First, let $G_{0}=[\begin{array}{cc} 1 & 1\\ 0 & 1 \end{array}]$. Then, for *j* = 1, …, *q*, let $G_{j}=[\begin{array}{cc} \text{cos}(\omega_{j}) & \text{sin}(\omega_{j})\\ -\text{sin}(\omega_{j}) & \text{cos}(\omega_{j}) \end{array}]$. Finally, the *F* and *G* matrices are as follows:

$$\begin{array}{c} \begin{array}{ccc} F & = & I_{6\times 6}⊗{\left(1,0,1,0,...,1,0\right)}_{(2+2q)\times 1}\\ G & = & I_{6\times 6}⊗\text{blockdiag}{\left(G_{0},G_{1},...,G_{q}\right)}_{\left(2+2q)\times (2+2q\right)}. \end{array} \end{array}$$ (2)

Here, the blockdiag() function creates a block diagonal matrix such that the ordering of the arguments from left to right corresponds to the order that the arguments appear in the resulting matrix from top left to bottom right. If *q* = 6, then we must define *ξ*_*c*,6_(*t*) = −*ξ*_*c*,6_(*t* − 1). We would also need a 1 × 1 matrix *G*_6_ = −1, and we would have to set *F* to be *F* = *I*_6×6_ ⊗ (1, 0, 1, 0, …, 1, 0, 1)_13×1_. This implies that *θ*_*t*_ is then of dimension (2 + 2(6) − 1)(6) × 1, or 78 × 1. This is the final model that we will use for our analysis.

### Estimation

We take a Bayesian approach to estimation for two main reasons. The first reason is that we wish to do inference, including uncertainty, on the evolution and error correlations, as this allows us to assess whether or not crime types are correlated. The second reason is that this also allows us to account for uncertainty in hyperparameters like variances and correlations when we try to estimate uncertainties in the latent states.

We chose to use independent half-Cauchy, *Ca*^+^(0, 1), priors on the evolution and residual standard deviations, and we then put independent priors of the form *p*(Ω|*v*) ∝ |Ω|^*v*−1^ (*v* > 0) on the correlation matrices for the residual and evolution vectors, Ω_*ϵ*_ and Ω_*δ*_, respectively [17]. With *v* = 1, this prior corresponds to a uniform distribution over correlation matrices, and thus we chose this prior with *v* = 1 in order to express our a priori lack of knoweldge over what the correlation structure might be. This prior, proposed by [17] is referred to as the *LKJ* distribution. One feature of this distribution, however, is that the implied marginal priors on any given correlation is that of a linearly transformed $\text{Beta}(\frac{d}{2},\frac{d}{2})$ distribution on (−1, 1), where *d* × *d* is the dimension of the correlation matrix [17]. This means that this prior does enforce some shrinkage towards zero for our correlations. A primary goal of our analysis is the detection of correlations between crimes, and so our prior can be thought of as slightly conservative in this setting. We note that our implied prior for the covariance matrix has a distrinct advantage over the common inverse Wishart distribution in that when the true marginal variances are close to zero, the inverse Wishart distribution has a tendency to bias the posterior variances upward. This also has the effect of biasing the correlations to zero [18]. Also, for a given covariance matrix Σ with diagonal elements $\sigma_{1}^{2},\ldots ,\sigma_{6}^{2}$ and corresponding correlation matrix Ω, the following transformation holds: Σ = diag(*σ*_1_, *σ*_2_, …, *σ*_6_)Ωdiag(*σ*_1_, *σ*_2_, …, *σ*_6_). Lastly, we put a diffuse Gaussian prior on the initial state, specifically, *p*(*θ*_0_) = *N*_6(2+2*q*)_(0, 10^7^ *I*) where *I* is the identity matrix. Thus, the full posterior distribution can be written out as $p\left(\theta ,\Sigma_{\epsilon },\Sigma_{\delta }|Y\right)\propto [\prod_{t=1}^{T}N\left(Y_{t};F\theta_{t},\Sigma_{\epsilon }\right)N\left(\theta_{t};G\theta_{t-1},\Sigma_{\delta }\right)]N\left(\theta_{0};0,10^{7}I\right)$.

To perform inference in our model, we used the package rstan through the statistical software environment R to do a Bayesian analysis via Markov chain Monte Carlo (MCMC) [19, 20]. We used the default variant of Hamiltonian Monte Carlo (HMC), a type of MCMC, implemented in Stan called the No-U-Turn Sampler to efficiently sample from the posterior distribution *p*(Σ_*ϵ*_, Σ_*δ*_|*Y*) [21]. In order to target this marginal posterior distribution, Stan computes the integral ∫ *p*(*θ*, Σ_*ϵ*_, Σ_*δ*_|*Y*)*dθ* using the Kalman filter [22]. Here, *Y* denotes all observed data obtained up to time *T*. Given these samples, the R package dlm [23] was used to get samples from *p*(*θ*|Σ_*ϵ*_, Σ_*δ*_, *Y*) through the forward filtering backward sampling (FFBS) algorithm [24, 25].

We used the potential scale reduction factor of Gelman and Rubin [26] to assess convergence of the three chains. Additionally, we calculated the number of effective samples for each sampled parameter to ensure reasonable accuracy in the tails of the posterior distribution.

## Analysis

We included four harmonics in the model as inference on the correlations between trends, our main concern in this analysis, was effectively identical to that which would have been based on including all six harmonics, and density plots of the evolution, error, and partial evolution correlations for the six harmonic model can be found in the supplementary information as S1–S3 Figs. In addition, we calculated the deviance information criterion (DIC), for both the six and four harmonic models. Much like the Akaike Information Criterion (AIC), DIC is a model fit criterion that penalizes models with many parameters, and models with lower values are preferred to ones with high values. Our DIC calculation results in values of approximately 182.29 for the six harmonic model and −141.03 for the four harmonic model.

Three chains consisting of 1000 warm-up iterations and 4000 inferential iterations (5000 total iterations per chain) were run. Effective sample sizes were all in excess of 5000, and the upper 95% confidence limits for all Gelman-Rubin diagnostics were < 1.01. Hence, following rough guidelines given in [27], starting values did not appear to impact convergence, and the effective samples were large enough to ensure accurate inference.

Additionally, as an empirical check for the validity of the distributional assumption on the observed data, estimated residual errors were calculated by subtracting the posterior time series means from the data, and the energy test for multivariate normality was performed [28], resulting in a p-value of approximately 0.194. Thus, we proceed assuming that our data is approximately normal.

### Results

**Fig 2:**
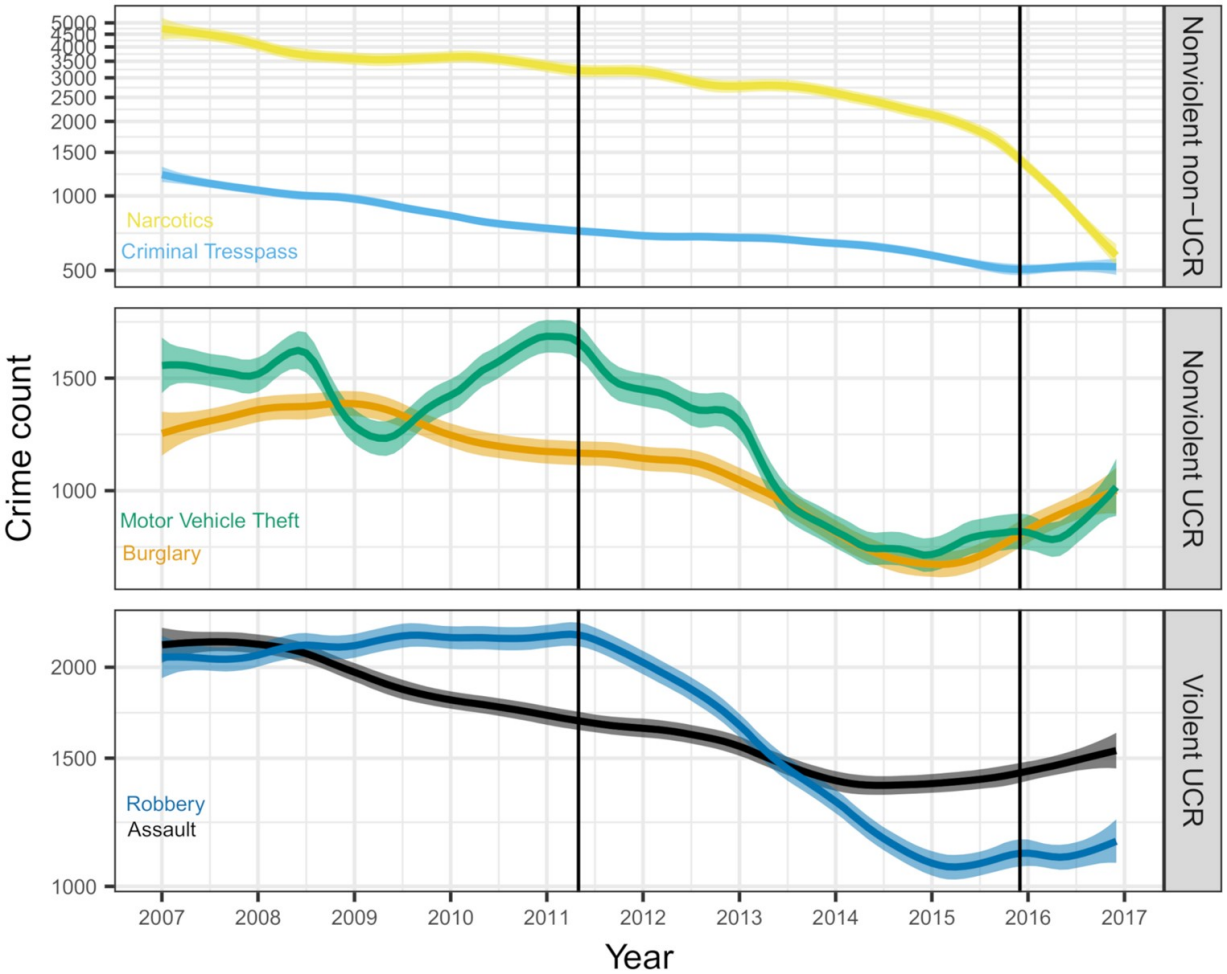
Smoothed time series means and 95% pointwise credible intervals for monthly crime counts of robberies, burglaries, assaults, motor vehicle thefts, criminal trespasses, and narcotics crimes in Chicago from January 2007 to December 2016. Changes to the police superintendent are indicated (vertical lines).

Fig 2 shows the estimated posterior means and pointwise 95% credible intervals for the smoothed time series means. The extracted trends clearly show substantial decreases in crime over this time period, though, as noted previously, there are noticeable differences in the shapes of the time series trajectories. Criminal trespasses, for example, show a reliable decline over the decade. Alternatively, robberies appeared to be on a slight upward trajectory from 2007 to roughly 2011, at which point some large change in reporting or criminal activity took place, leading to a roughly 50% drop in reported robberies over the subsequent four or five years.

Based on the smoothed time series means, it is clear that some trends are more similar than others, but correlations in the evolutions give a way of probabilistically quantifying how likely it is that any two given crimes co-evolve in a dependent fashion. We have approximate samples from our posterior distribution allowing us to estimate the probability that two crimes are positively correlated by computing the proportion of MCMC samples of the relevant correlation parameter that are positive. Mathematically, let $\rho_{\delta }^{\left(i,j\right)}$ be the (*i*, *j*)-th evolution correlation. Then an estimate of the probability that the evolutions between crime type *i* and crime type *j* are positively correlated is given by $P\left(\rho_{\delta }^{\left(i,j\right)}>0|Y\right)\approx \frac{1}{12000}\sum_{k=1}^{12000}I[\rho_{\delta }^{{\left(i,j\right)}^{\left(k\right)}}>0]$, where $\rho_{\delta }^{{\left(i,j\right)}^{\left(k\right)}}$ is the *k*-th MCMC sample for the evolution correlation between crime type *i* and crime type *j*. Table 2 gives estimates of the posterior probabilities that the evolution correlation between any two crime types is positive (above the diagonal), as well as lower bounds for one-sided 95% credible intervals of these correlations (below the diagonal). The four largest estimated probabilities are all fairly similar and exceed 0.8. These correspond to correlations in the trends between robberies and burglaries, robberies and motor vehicle thefts, assaults and burglaries, and assaults and motor vehicle thefts. Interestingly, these crimes are exactly the four crimes in our dataset that are tracked in the UCR. The next largest estimated probability is approximately 0.61, which is substantially smaller. Also, there are a number of small estimated probabilities that are near 0.2. Three estimated probabilities are below 0.2, implying estimated probabilities of negative evolution correlations in excess of 0.8. These estimates correspond to narcotics and robberies, narcotics and burglaries, and criminal trespasses and narcotics. Thus, we find some evidence of positive trend associations with robberies, assaults, burglaries, and motor vehicle thefts, but perhaps these crimes are inversely related to narcotics crimes. There are not any noteworthy differences in the posterior correlations either within or between violent and nonviolent crimes in our dataset.

**Table 2:** Estimated posterior probabilities of positive evolution correlations (above the diagonal), and lower bounds for one sided 95% credible intervals (below the diagonal) for monthly aggregated robberies, burglaries, assaults, motor vehicle thefts, criminal trespasses, and narcotics crimes in Chicago from January 2007 through December 2016.

	Robbery	Burglary	Assault	Narcotics	MVT	Trespass
Robbery	-	0.87	0.61	0.18	0.86	0.22
Burglary	-0.16	-	0.83	0.18	0.27	0.61
Assault	-0.42	-0.22	-	0.28	0.82	0.39
Narcotics	-0.71	-0.71	-0.67	-	0.28	0.19
MVT	-0.20	-0.65	-0.27	-0.66	-	0.24
Trespass	-0.71	-0.42	-0.60	-0.72	-0.73	-

**Fig 3:**
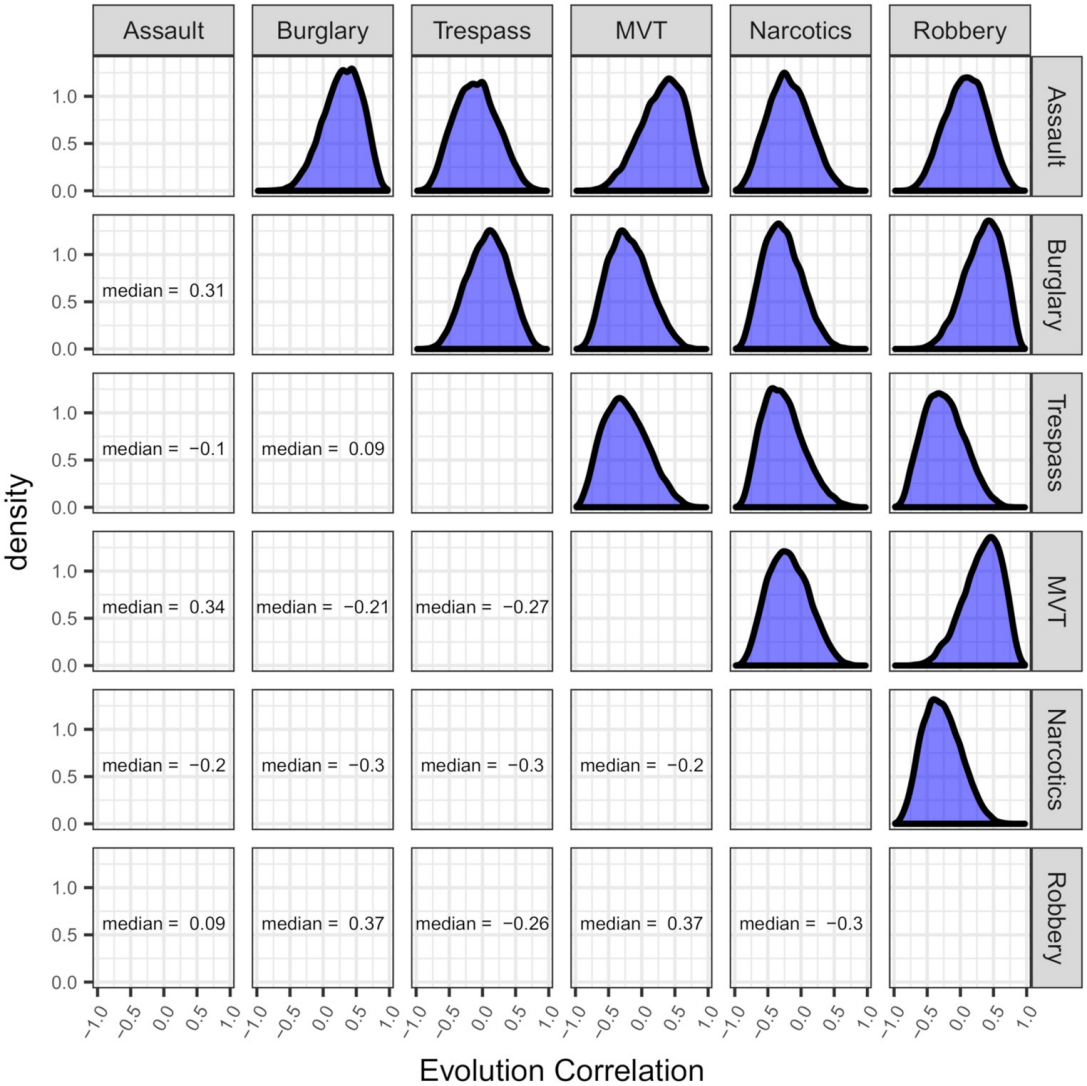
Smoothed estimated posterior evolution correlation densities for monthly aggregated robberies, burglaries, assaults, motor vehicle thefts, criminal trespasses, and narcotics crimes in Chicago from January 2007 through December 2016.

For a more complete picture of these posterior correlations, Fig 3 gives a matrix of estimated posterior densities as well as estimated posterior medians for the correlations of any two given crime types. The estimated posteriors seem to be fairly wide, indicating a relatively large degree in uncertainty as to what the associations between crime trends over our period of interest might actually be. Posterior modes of correlations are at most near 0.4, and at the least they are near −0.3.

**Fig 4:**
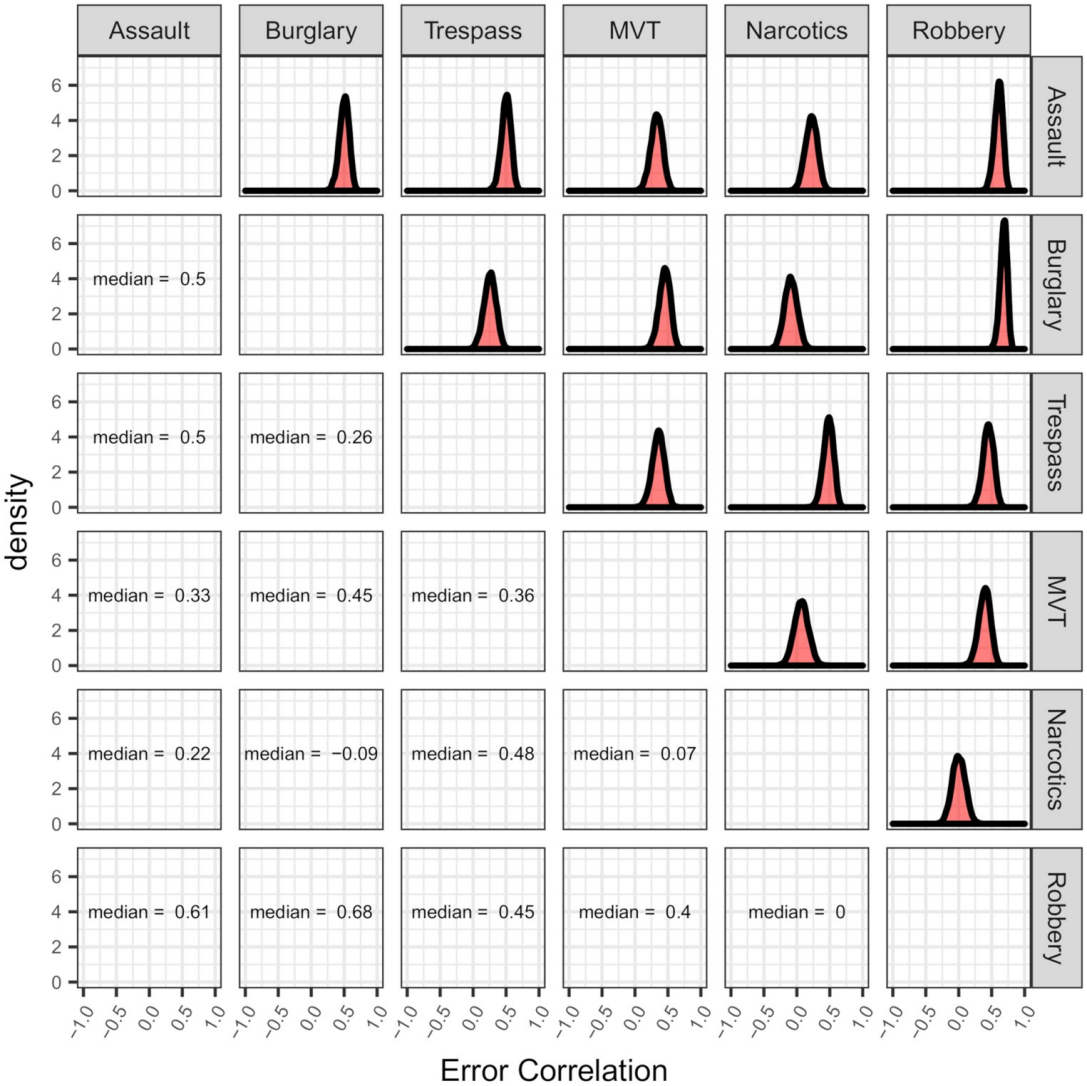
Smoothed estimated posterior residual error correlation densities for monthly aggregated robberies, burglaries, assaults, motor vehicle thefts, criminal trespasses, and narcotics crimes in Chicago from January 2007 through December 2016.

By contrast, we can examine similar density plots (Fig 4) and estimated posterior probabilities (Table 3) for the residual error correlations between crime types. Here, the estimated posterior distributions suggests much stronger evidence that there is a moderate degree of positive association between most of the crime types shown here. In fact, the only estimated probabilities of positive correlation that are below 0.9 are for motor vehicle thefts and narcotics, narcotics and burglaries, and assaults and robberies. However, in the case of narcotics and burglaries, there is some evidence that they are negatively associated on a monthly basis, as this probability is approximately 1 − 0.179 = 0.821.

**Table 3:** Estimated posterior probabilities of positive error correlations (above the diagonal), and lower bounds for one-sided 95% credible intervals (below the diagonal) for monthly aggregated robberies, burglaries, assaults, motor vehicle thefts, criminal trespasses, and narcotics crimes in Chicago from January 2007 through December 2016.

	Robbery	Burglary	Assault	Narcotics	MVT	Trespass
Robbery	-	1.00	1.00	0.50	1.00	1.00
Burglary	0.58	-	1.00	0.18	1.00	1.00
Assault	0.49	0.37	-	0.99	1.00	1.00
Narcotics	-0.17	-0.25	0.06	-	0.74	1.00
MVT	0.24	0.30	0.17	-0.11	-	1.00
Trespass	0.30	0.01	0.37	0.34	0.19	-

Again, for a more complete picture of the error correlations, Fig 4 gives the estimated posterior error correlation densities as well as estimated posterior medians. The estimated posterior distributions here look reasonably symmetric, and the posterior modes tend to vary a bit more than in the case of the evolution correlations, even for crime pairs with high probabilities of being positive. Such a phenomenon can also be seen by looking at the lower 95% credible bounds in Table 3. For example, an estimate of the median posterior correlation between robberies and burglaries is around 0.684, whereas another crime pair with high probability of being positive, burglaries and criminal trespasses, has an estimate of the median correlation of approximately 0.259.

In a temporal setting like ours, we can also examine conditional dependence between crime types by simply looking at the posterior distribution of components of inverted covariance matrices. Table 4 gives estimates of the probability that the partial correlation between the evolutions of two crime types is positive. The assumption that the states and data are Gaussian means that very large or very small probabilities correspond to evidence in favor of conditionally dependent trends. Interestingly, crime pairs with high probabilities of positive marginal correlation often have high probabilities of negative conditional correlation. Robberies and burglaries, for example, have a probability of negative partial correlation of 1 − 0.088 = 0.912. Assaults and burglaries also have high probability of negative partial correlation 1 − 0.097 = 0.903. Criminal trespasses and narcotics crimes and motor vehicle thefts and burglaries both have probabilities of positive partial correlations of around 0.89. Additionally, assaults and robberies have a somewhat high probability of having positive partial correlation at around 0.83.

**Table 4:** Estimated posterior probabilities of positive evolution partial correlations (above the diagonal), and lower bounds for one-sided 95% credible intervals (below the diagonal) for monthly aggregated robberies, burglaries, assaults, motor vehicle thefts, criminal trespasses, and narcotics crimes in Chicago from January 2007 through December 2016.

	Robbery	Burglary	Assault	Narcotics	MVT	Trespass
Robbery	-	0.09	0.83	0.70	0.17	0.80
Burglary	-0.98	-	0.10	0.58	0.89	0.38
Assault	-0.39	-0.98	-	0.64	0.15	0.70
Narcotics	-0.62	-0.82	-0.70	-	0.62	0.89
MVT	-0.97	-0.29	-0.98	-0.83	-	0.55
Trespass	-0.49	-0.93	-0.64	-0.28	-0.90	-

**Fig 5:**
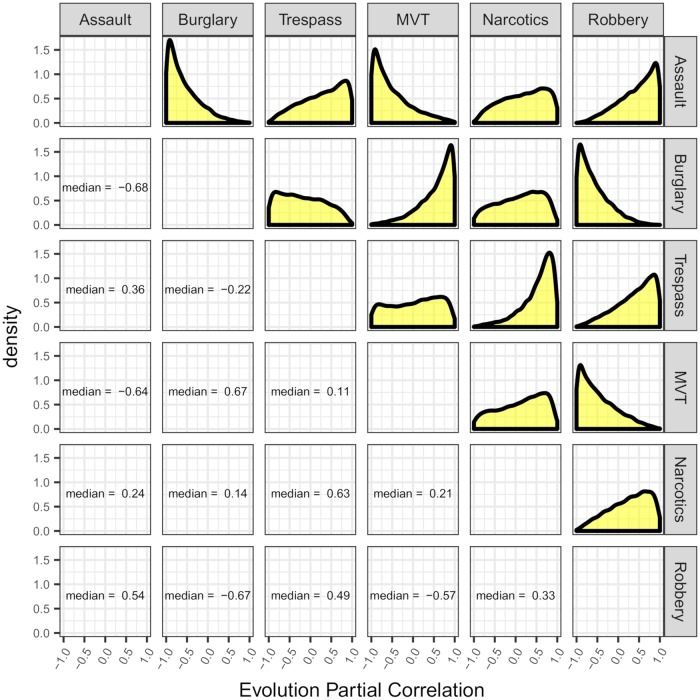
Smoothed estimated posterior partial evolution correlation densities and estimated posterior medians for monthly aggregated robberies, burglaries, assaults, motor vehicle thefts, criminal trespasses, and narcotics crimes in Chicago from January 2007 through December 2016.

A noticeable pattern emerges by looking at the estimated posterior partial correlation densities in Fig 5. Here we see several obviously peaked and skewed densities, some mostly flat densities, and few inbetween. Interestingly, the peaked densities that are skewed left (indicating high probabilities of positive partial correlation) are seen only between pairs of crimes which both occur in one of the three crime categorizations we’ve used. Similarly, the peaked densities that are skewed right (indicating high probabilities of negative partial correlation) are seen only between pairs of crimes for which each crime is categorized differently. In particular, these strong negative conditional associations are seen between violent and nonviolent UCR crimes.

## Discussion

We utilized dynamic linear models to model crime trends, and we inferred dependencies between trends of different crime types. We found that the only four crime pairs for which the estimated posterior probabilities of positive evolution correlation exceeded 0.8 were all counted in the FBI’s UCR, and every UCR-tracked crime that we modeled showed up in at least one crime pair where this probability exceeded 0.8. An even more obvious pattern emerged when we analyzed the evolution partial correlations. Some partial correlation distributions are very clearly peaked and concentrated near −1 or 1, others are very flat, and there are very few in between. Highly positive, peaked partial correlation distributions occur for burglaries and motor vehicle thefts, assaults and robberies, and narcotics crimes and criminal trespasses. In addition, highly negative, peaked partial correlation distributions occur for burglaries and assaults, burglaries and robberies, assaults and motor vehicle thefts, and robberies and motor vehicle thefts. Thus, we found evidence that there is considerable shared information between different types of crimes. Moreover, high positive conditional associations appear almost exclusively within each of the crime categories we considered, while high negative conditional associations appear almost exclusively between categories. Thus, forces that affect crime or its reporting may do so differently depending on the nature of the crime. There was no noticeable consistent pattern when comparing trend correlations between and within violent and nonviolent crimes. Further, based on our correlation analyses, we found little evidence of the suggested misreporting of burglaries as criminal trespasses. In contrast to the evolution correlations, we found that the correlations between the residual errors to be almost universally positively correlated with extremely high probability. These correlations carry different interpretations, and, in general, either or both may be of interest when comparing multiple time series.

The analysis of [11] found support for the “broken windows theory” by finding positive correlations between thefts (considered a mild crime) and robberies and between thefts and burglaries on a quarterly scale. [12] examined correlations between mild and serious crimes and found that, on the scale of days, there was little evidence of positive correlations between crime types and hence did not find support for the “broken window theory”. Similarly, [13] found little evidence for the “broken windows theory” on the scale of weeks when comparing many of the same serious crimes considered in our analysis. Other than the residual error correlations, which were positive for nearly all pairs of crimes, our analysis found little in the way of positive correlations between crimes not included in the UCR and crimes included in the UCR, though our analysis also sought to examine correlations in the form of contemporaneous trend deviations between crimes as opposed to correlations between different crimes at different lags. Contemporaneous trend deviations are sensible to examine if the hypothesis to be assessed is that serious crimes are being misreported as mild crimes, as one would not expect, for example, burglaries in one month to be reported as criminal trespasses in a different month.

Many studies, including those of [11–13] allude to other factors that may affect crime itself, rather than crime reporting, such as weather, economic conditions, and police activity. Most such factors should arguably correlate positively with all crime. This may one reason why the residual error correlations seemed almost to be universally positive. However, the fact that we see such distinctly different partial evolution correlations when looking within a given class of crime versus between a two given classes of crime seems to suggest some additional factor at play. Supposing that the observed correlations in these data are indicative of correlations in actual crimes committed, the patterns in partial correlations observed here appear consistent with observations made in [29] that burglaries and robberies are substitutes while burglaries and auto thefts are complementary. However, given that our initial analysis objective was not to test for these types of effects, this observation should not be taken as confirmatory.

In a spatial setting, [10] proposed a way to study conditional linear associations between crime types using graphical models, where nodes in a graph represent one component of a multivariate point process, and edges represent non-zero conditional linear associations. His method, dubbed the spatial dependence graph model, uses the partial spectral coherence in order to calculate the linear relationship between components of a multivariate point process after the elimination of the linear effect of all other components. Eckardt applied his method to crime in London and, like in our analysis, found that a small number of subgraphs emerged. All crimes included in our model, with the exception of criminal trespasses, were either also modeled by Eckardt, or there was a close analog. Taking this into consideration, both we and Eckardt found evidence for conditional dependencies between burglaries, robberies, and motor vehicle thefts. However, we found evidence of temporal conditional dependence between assaults and burglaries and between assaults and motor vehicle thefts, whereas Eckardt found no spatial conditional linear association between the London analog to assaults and any other crime.

One caveat to DLMs is the required assumption that the data be Gaussian. This restricts the usage of DLMs to situations where crime counts are sufficiently large in any given time period. Often this is not a problem, but the Gaussian assumption can be difficult to justify for infrequent crimes or crimes at small time scales. The assumption of normality can be replaced by the more natural assumption that crime counts follow a Poisson distribution. This would enable much of the same analysis that was done here, but one could also consider much less frequent crimes at shorter time scales. This generalization takes us to the realm of dynamic generalized linear models (DGLMs): a set of time series models where the data model is non-Gaussian. A Bayesian analysis in these models is also possible, but computation becomes less straightforward. Nevertheless, state-space models such as DLMs and DGLMs are interesting and sensible methods for the modeling of crime and the analysis of its trends.

## Supporting information

S1 Fig Evolution correlation posterior densities (six harmonics). (TIF)

S2 Fig Error correlation posterior densities (six harmonics). (TIF)

S3 Fig Partial evolution correlation posterior densities (six harmonics). (TIF)
