# Supplementary figures and images for: Multivariate temporal modeling of crime with dynamic linear models
Source: PLoS One. 2019 Jul 3;14(7):e0218375. doi: 10.1371/journal.pone.0218375 (PMC6608923; doi:10.1371/journal.pone.0218375)

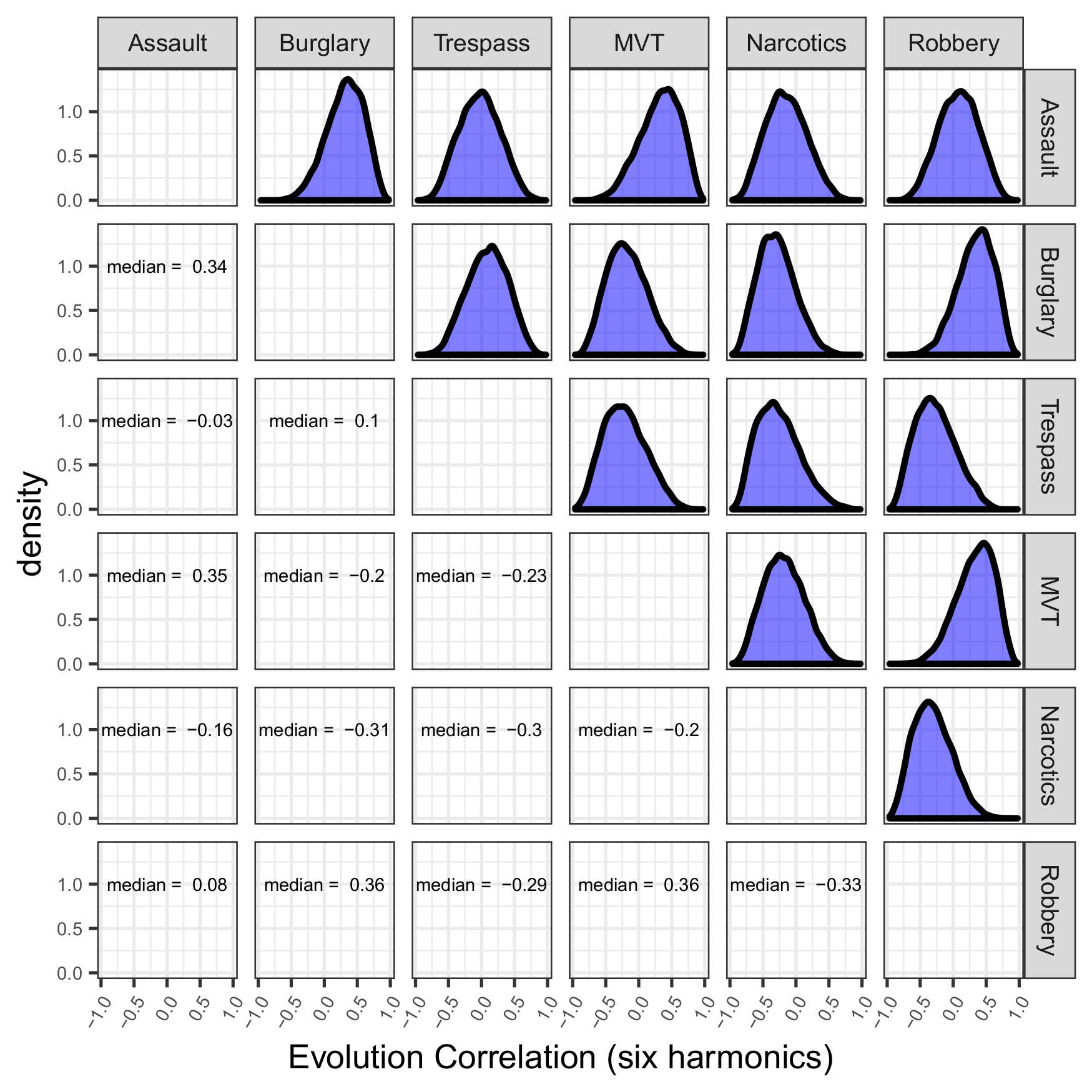

Supplement: S1 Fig — (TIF) [file pone.0218375.s001.tif]

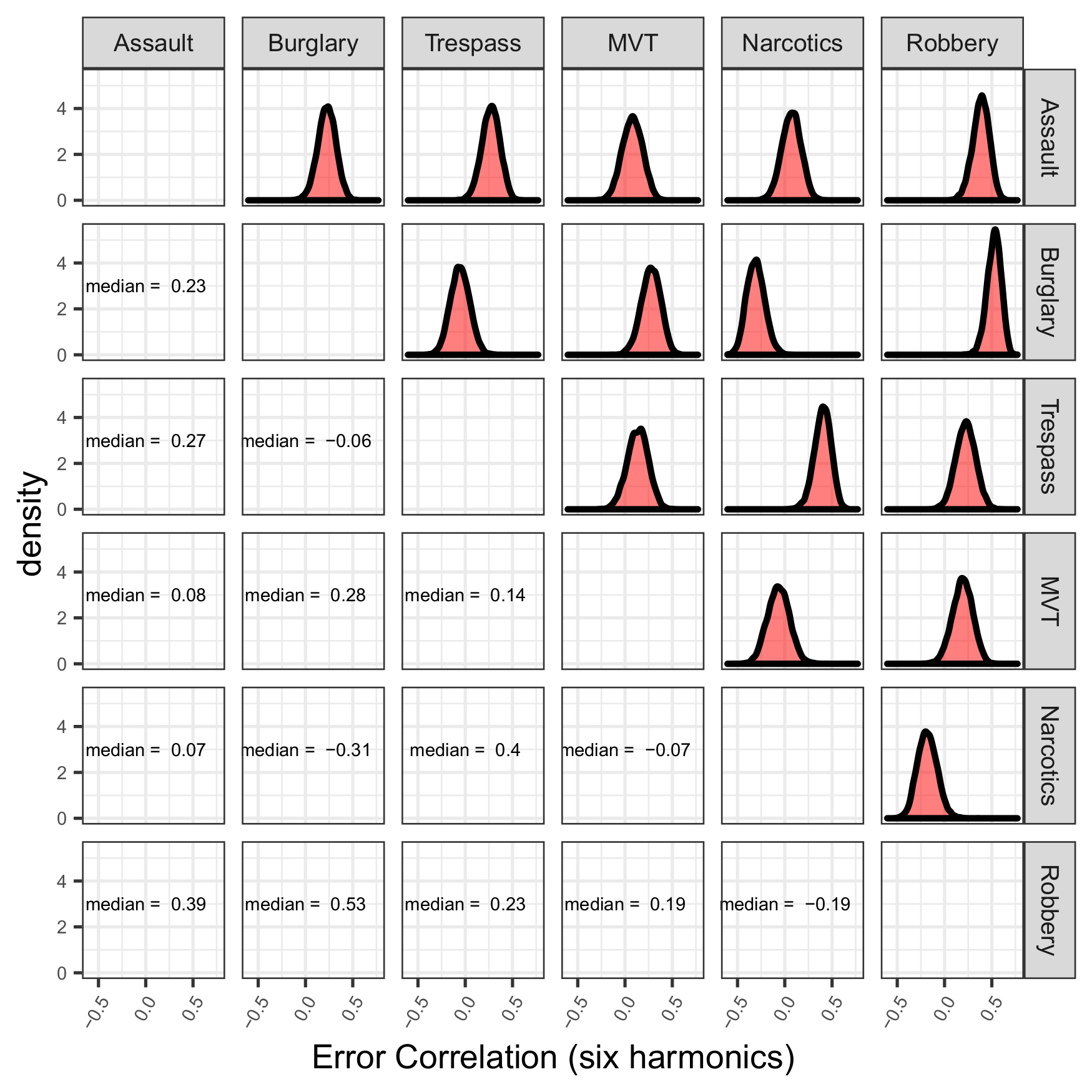

Supplement: S2 Fig — (TIF) [file pone.0218375.s002.tif]

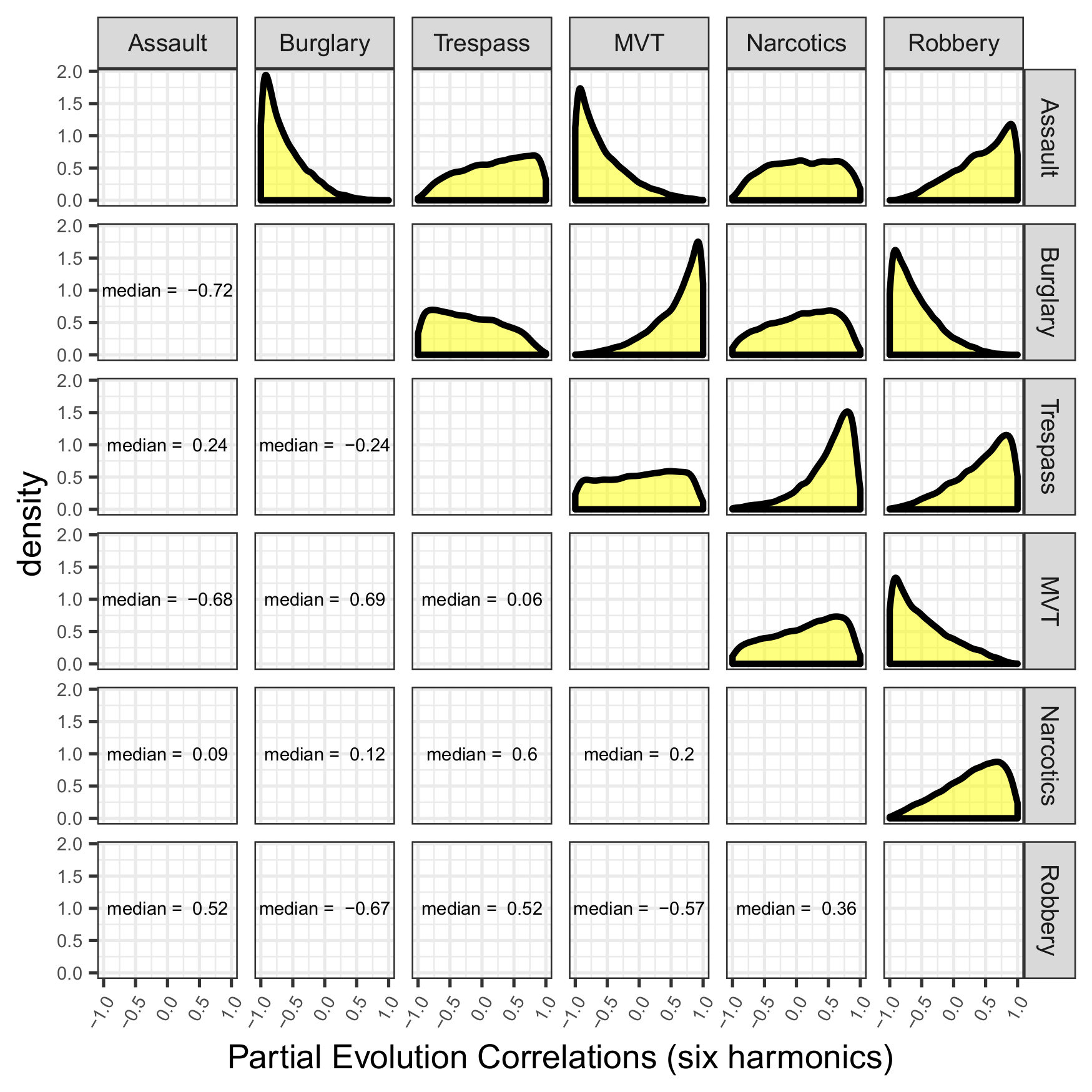

Supplement: S3 Fig — (TIF) [file pone.0218375.s003.tif]
